# Supplementary material for: Extracellular vesicles could be a putative posttranscriptional regulatory mechanism that shapes intracellular RNA levels in Plasmodium falciparum
Source: Nat Commun. 2023 Oct 13;14:6447. doi: 10.1038/s41467-023-42103-x (PMC10575976; doi:10.1038/s41467-023-42103-x)
Supplement: Supplementary file 1 — Supplementary Information [file 41467_2023_42103_MOESM1_ESM.pdf]

Supplementary figures for

Title: Extracellular vesicles could be a putative posttranscriptional regulatory mechanism that shapes intracellular RNA levels in *Plasmodium falciparum*

Authors: Mwikali Kioko, Alena Pance, Shaban Mwangi, David Goulding, Alison Kemp, Martin Rono, Lynette Isabella Ochola-Oyier, Pete C. Bull, Philip Bejon, Julian C. Rayner, Abdirahman I. Abdi

Corresponding author aabdi@kemri-wellcome.org

This word file includes:

Supplementary Fig 1 to 6

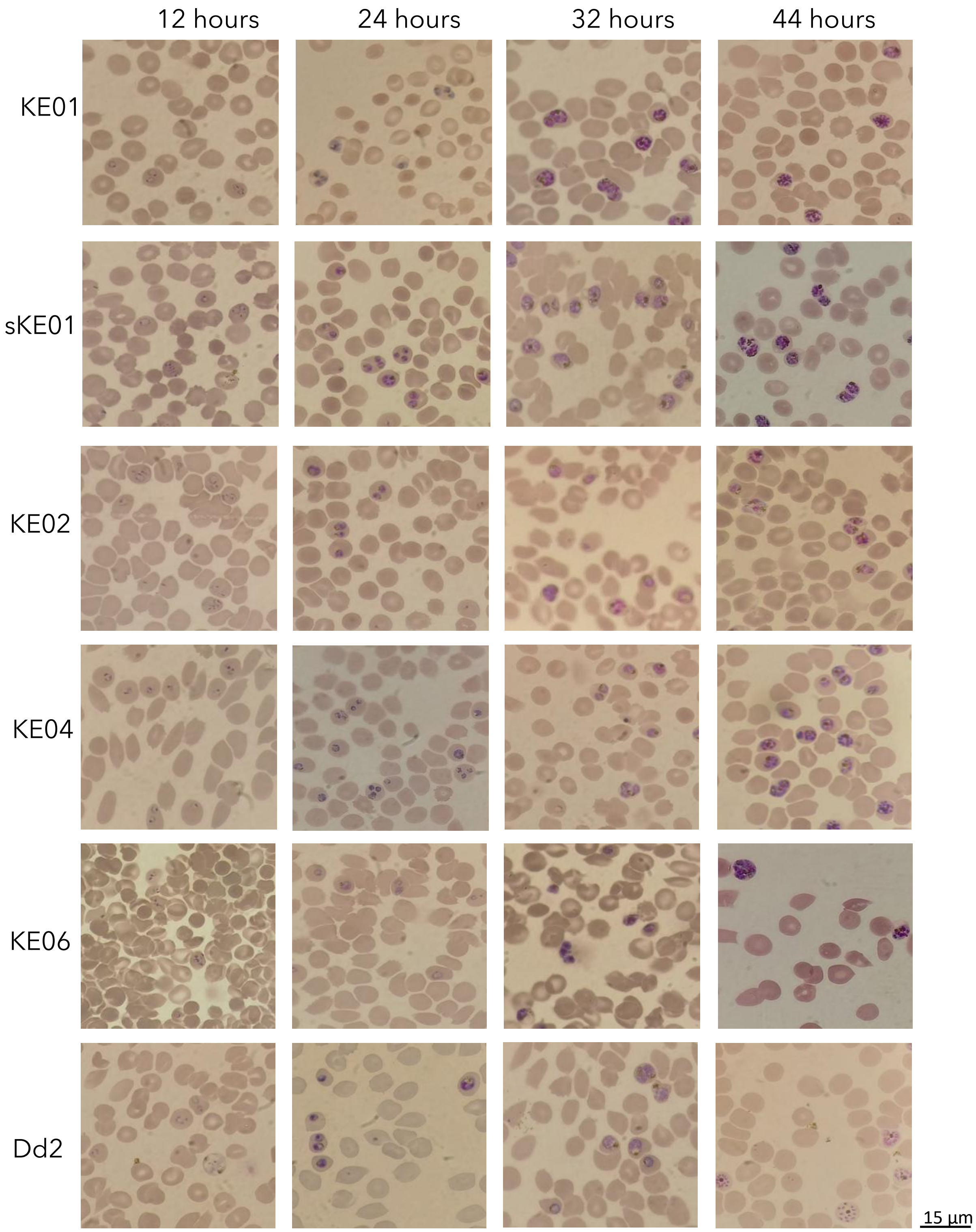

Supplementary Fig. 1 Giemsa smears of the asexual cultures obtained from four time points

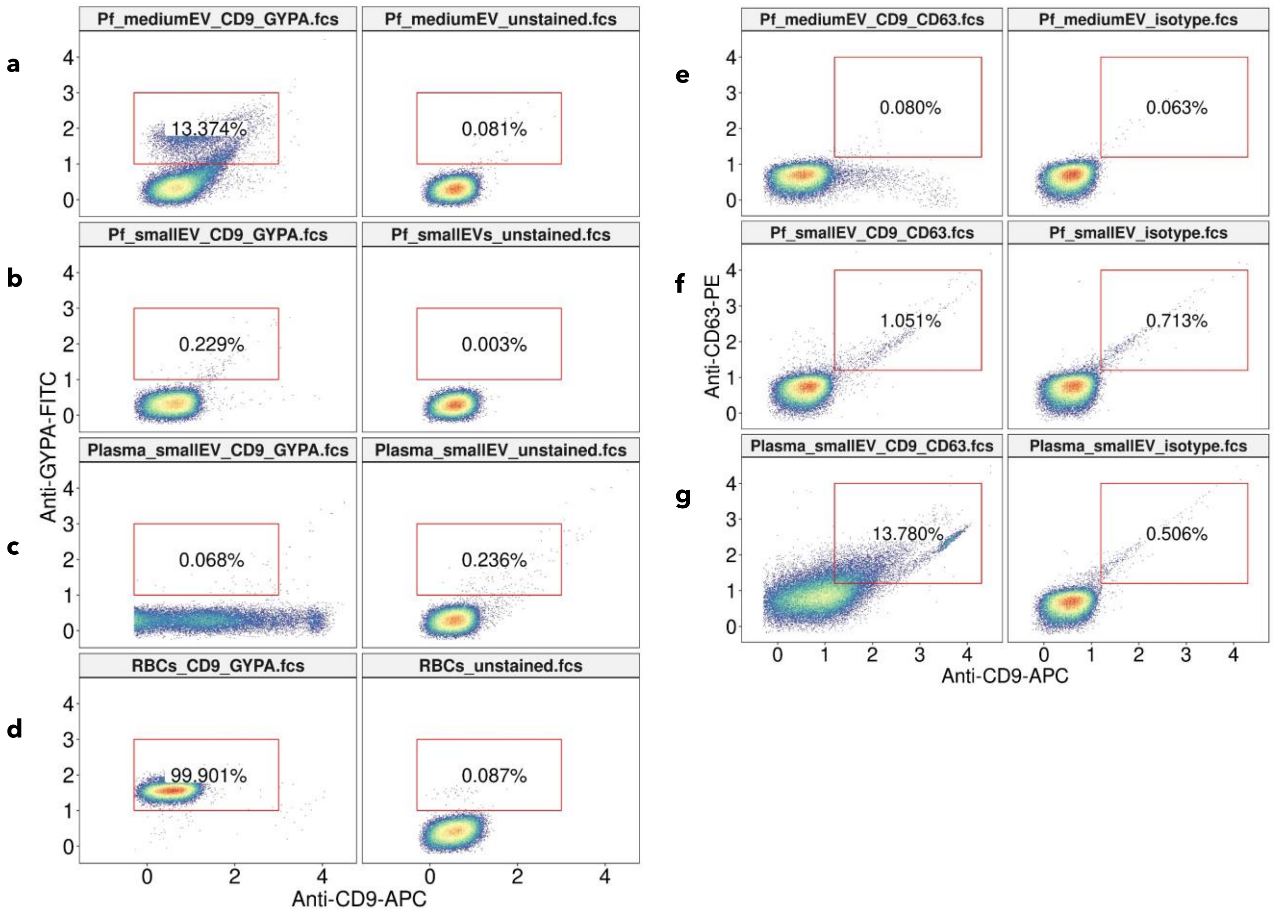

Supplementary Fig. 2: Bead-assisted EV flow cytometry

EVs were isolated from plasma using nanofiltration and ultracentrifugation.

a-d The pellets of *Pf* medium EV, *Pf* small EV, plasma small EV and RBCs (left panel), respectively were analysed by bead-assisted flow cytometry using anti-GYPA-FITC and anti-CD9-APC. EVs were first coupled to latex aldehyde-sulfate beads. Only the proportion positive for GYPA was gated. Unstained samples were used as negative controls (right panel). e-g The pellets of *Pf* medium EV (formerly called microvesicles), *Pf* small EV and plasma small EVs (formerly called exosomes), respectively were analysed by bead-assisted flow cytometry using anti-CD63-PE and antiCD9-APC antibodies after coupling EVs with latex aldehyde –sulfate beads (left panel). Isotype antibodies were used as negative controls (right panel). The proportion positive for both CD9 and CD63 was gated.

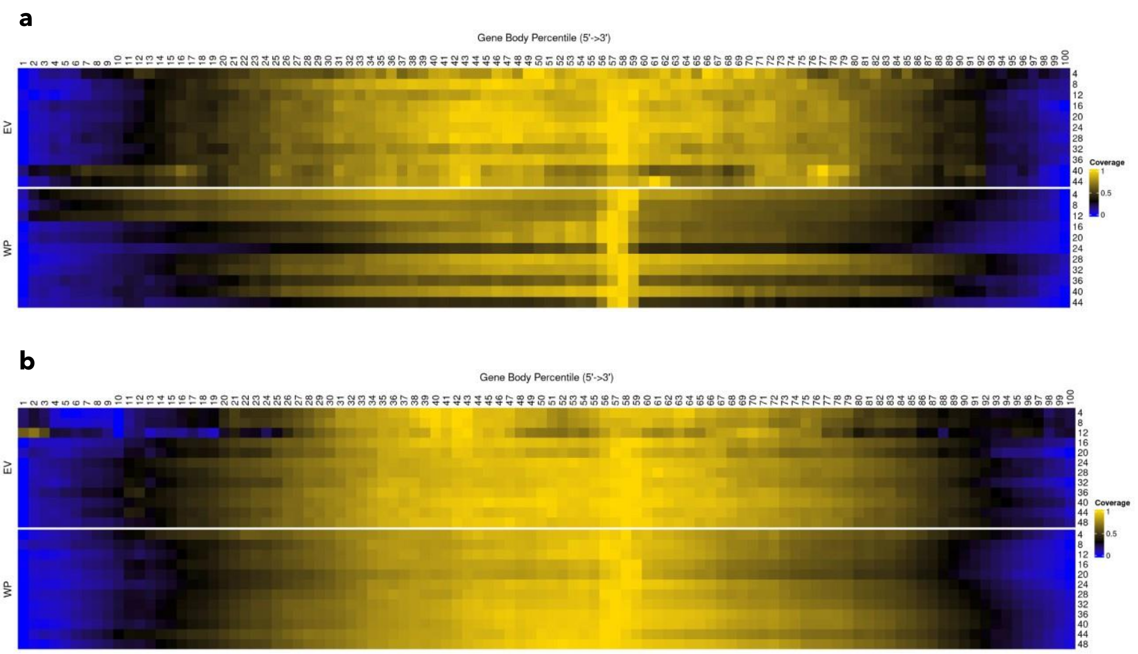

Supplementary Fig. 3: Comparison of gene body coverage between *Pf*EVs and WP
a and **b** Heatmap showing gene body coverage of RNAseq reads obtained from KE01 and KE02, respectively

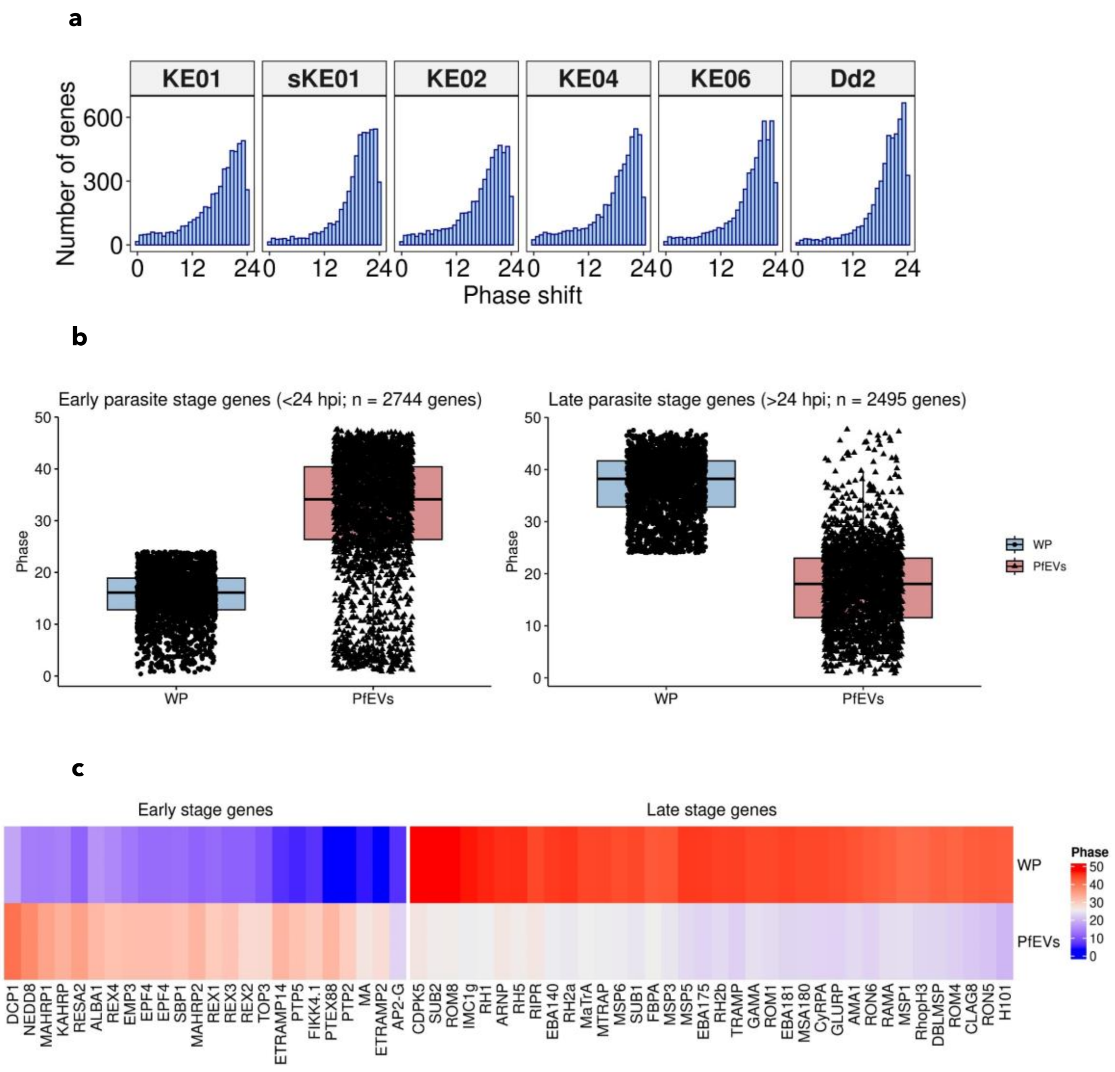

Supplementary Fig. 4: The phases of RNA abundance in *Pf*EVs are shifted compared to those of WP

a Histograms showing distribution of phase-shift of RNA secretion. **b** Early-stage transcripts are secreted via *Pf*EVs during late stages of the parasite and the vice versa is true. The centre lines represent the medians, limits represent the median ± interquartile range, and whiskers represent values 1.5 times above or below the 75th and 25th percentiles, respectively. **c** Heatmap demonstrating median phases in *Pf*EVs and WP for selected ring-stage and schizont genes.

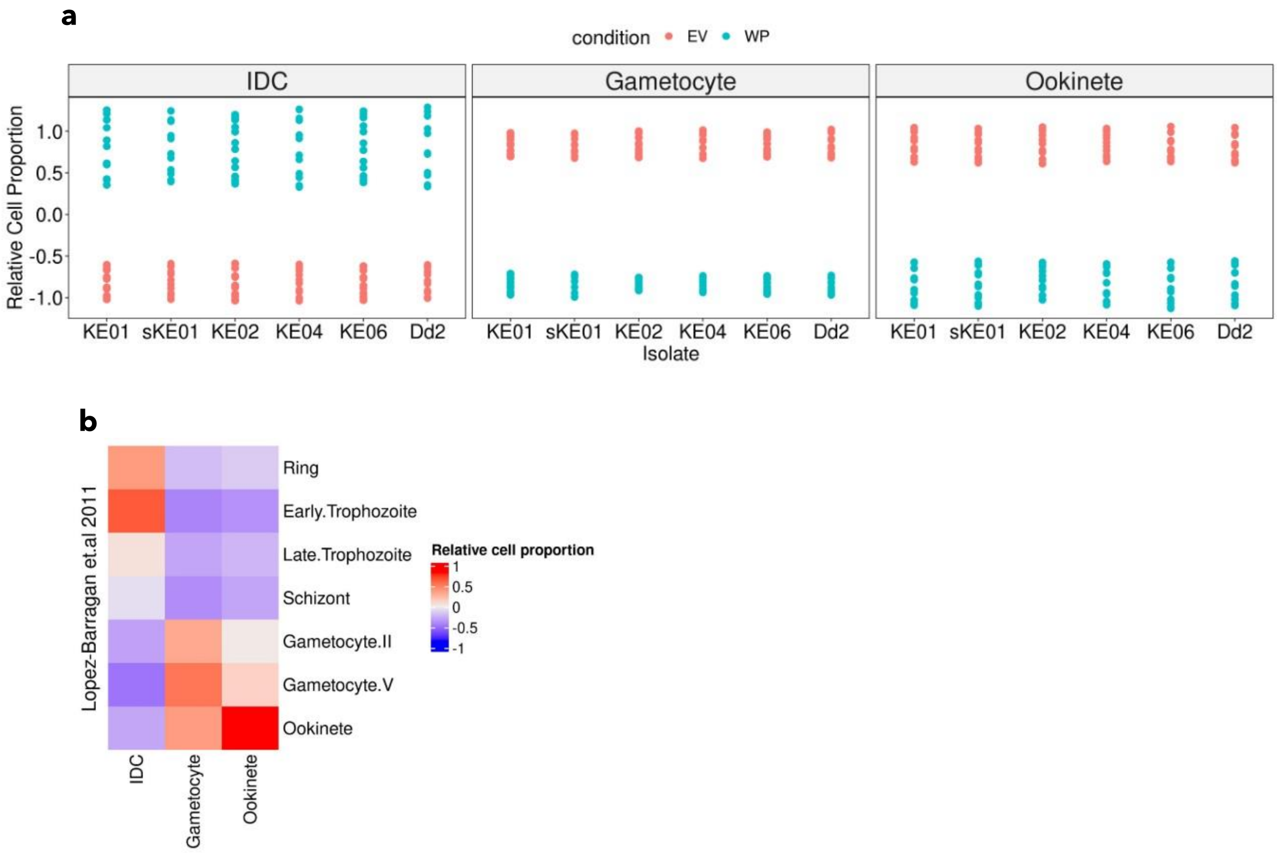

Supplementary Fig. 5 Relative deconvolution of RNA abundance in WP and *Pf*EVs using singular variable deconvolution

a Deconvolution of the RNAseq data using markers specific to IDC and two sexual stages (gametocyte and Ookinete) reveals that the WP have higher IDC specific RNA content and lower sexual stage RNA compared to the *Pf*EVs. **b** Validation of the RNA deconvolution method using Lopez-Barragan et.al 2011 dataset which is composed of four IDC stages (ring, early trophozoite, late trophozoite and schizont) and three sexual stages (gametocyte II, gametocyte V and Ookinete). Our deconvolution can accurately identify the dominant cell type using RNAseq data.

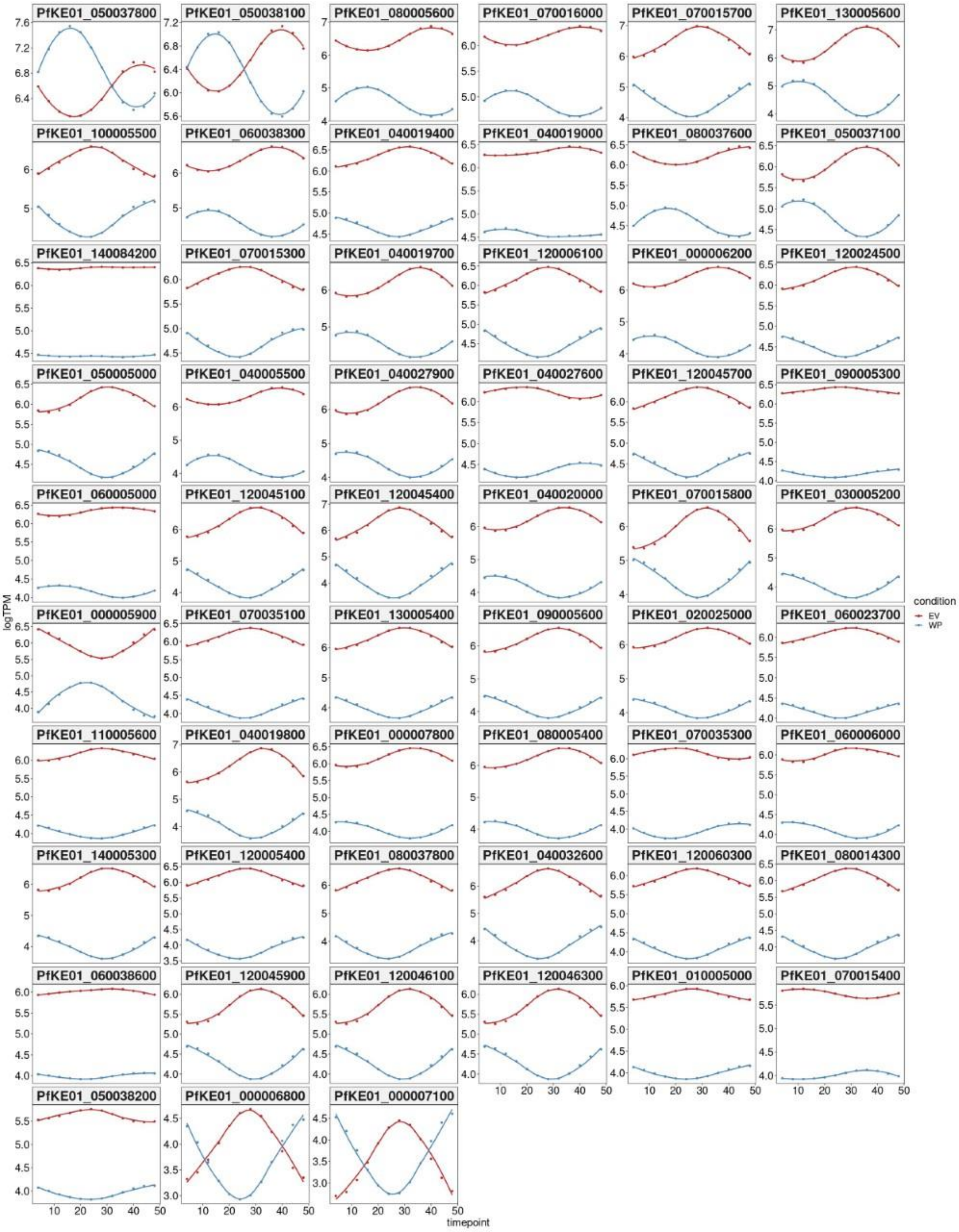

Supplementary Fig. 6 Comparisons of sKE01 var RNA abundance between *Pf*EVs and WP

The two dominant var gene, *Pf*KE01_050037800 and PfKE01_050038100 (top left), as the result of HBEC-5i selection, are transcribed in the WP at levels above those of secretion via *Pf*EVs in a higher proportion of parasites while the other vars are enriched in *Pf*EVs compared to the WP.
