# Supplementary material for: Extracellular vesicles could be a putative posttranscriptional regulatory mechanism that shapes intracellular RNA levels in Plasmodium falciparum
Source: Nat Commun. 2023 Oct 13;14:6447. doi: 10.1038/s41467-023-42103-x (PMC10575976; doi:10.1038/s41467-023-42103-x)
Supplement: Supplementary file 3 — Reporting Summary [file 41467_2023_42103_MOESM3_ESM.pdf]

nature portfolio

Corresponding author(s): Abdirahman I. Abdi

Last updated by author(s): Aug 23, 2023

Reporting Summary

Nature Portfolio wishes to improve the reproducibility of the work that we publish. This form provides structure for consistency and transparency in reporting. For further information on Nature Portfolio policies, see our Editorial Policies and the Editorial Policy Checklist.

Statistics

For all statistical analyses, confirm that the following items are present in the figure legend, table legend, main text, or Methods section.

n/a	Confirmed	
☐	☒	The exact sample size (n) for each experimental group/condition, given as a discrete number and unit of measurement
☐	☒	A statement on whether measurements were taken from distinct samples or whether the same sample was measured repeatedly
☐	☒	The statistical test(s) used AND whether they are one- or two-sided<br>*Only common tests should be described solely by name; describe more complex techniques in the Methods section.*
☐	☒	A description of all covariates tested
☐	☒	A description of any assumptions or corrections, such as tests of normality and adjustment for multiple comparisons
☐	☒	A full description of the statistical parameters including central tendency (e.g. means) or other basic estimates (e.g. regression coefficient) AND variation (e.g. standard deviation) or associated estimates of uncertainty (e.g. confidence intervals)
☐	☒	For null hypothesis testing, the test statistic (e.g. F, t, r) with confidence intervals, effect sizes, degrees of freedom and P value noted<br>*Give P values as exact values whenever suitable.*
☒	☐	For Bayesian analysis, information on the choice of priors and Markov chain Monte Carlo settings
☒	☐	For hierarchical and complex designs, identification of the appropriate level for tests and full reporting of outcomes
☐	☒	Estimates of effect sizes (e.g. Cohen's d, Pearson's r), indicating how they were calculated

Our web collection on statistics for biologists contains articles on many of the points above.

Software and code

Policy information about availability of computer code

Data collection	no software was used to collect data
Data analysis	The R statistical software (version 4.2.1) was used to perform all analyses. The RNA abundances were estimated using Kallisto R package and normalized for sequencing depth and length. RUVseq R package was used to adjust for unwanted variation while the CircaCompare R package was used to calculate rhythmic parameters. Heatmaps were constructed using ComplexHeatmap R package. No custom software was generated in this study

For manuscripts utilizing custom algorithms or software that are central to the research but not yet described in published literature, software must be made available to editors and reviewers. We strongly encourage code deposition in a community repository (e.g. GitHub). See the Nature Portfolio guidelines for submitting code & software for further information.

Data

Policy information about availability of data

All manuscripts must include a data availability statement. This statement should provide the following information, where applicable:

- Accession codes, unique identifiers, or web links for publicly available datasets
- A description of any restrictions on data availability
- For clinical datasets or third party data, please ensure that the statement adheres to our policy

The RNAseq data generated in this study is available in Gene Expression Ominbus under the accession number GSE241277. The Malaria Cell Atlas reused in this study is available in the European Nucleotide Archive (accession nos. ERP021229 and ERP124136).

Human research participants

Policy information about studies involving human research participants and Sex and Gender in Research.

Reporting on sex and gender	This information has not been collected because the study was based on laboratory cell lines rather than human participants
Population characteristics	This information was not collected because the study was based on laboratory cell lines rather than human particpants
Recruitment	This information is not available because the study was based on cell lines
Ethics oversight	The study was on in vitro culture adapted parasite lines rather than human participants but a written consent was obtained from the parent's of the children that donated the original clinical samples from which the parasite lines used in this study were adapted from. The study was approved by the relevant regulatory authority (KEMRI-SERU).

Note that full information on the approval of the study protocol must also be provided in the manuscript.

Field-specific reporting

Please select the one below that is the best fit for your research. If you are not sure, read the appropriate sections before making your selection.

[x] Life sciences [ ] Behavioural & social sciences [ ] Ecological, evolutionary & environmental sciences

For a reference copy of the document with all sections, see nature.com/documents/nr-reporting-summary-flat.pdf

Life sciences study design

All studies must disclose on these points even when the disclosure is negative.

Sample size	A sample size of 6 parasite lines was chosen. This is based on a previous study (https://bmcgenomics.biomedcentral.com/articles/10.1186/s12864-018-5257-x) which reported that six parasite isolates were adequate to detect biological signals.
Data exclusions	No data were excluded
Replication	We measured RNA from six parasite lines, which are equivalent to six biological replicates. We did not have technical replicates because large-scale culturing was required to harvest culture-conditioned media in one parasite cycle (i.e. approximately 3 Litres per parasite isolate).
Randomization	Randomization was not done. The Kenyan parasite isolates were chosen because their genomes have been sequenced and are currently been assembled in a different study and the genome of one of them (KE01) is available in plasmodb.
Blinding	Blinding was not considered essential in this study. The authors who generated the data were aware of the identities of the parasite isolates and we do not think this has in anyway affected the study.

Reporting for specific materials, systems and methods

We require information from authors about some types of materials, experimental systems and methods used in many studies. Here, indicate whether each material, system or method listed is relevant to your study. If you are not sure if a list item applies to your research, read the appropriate section before selecting a response.

Materials & experimental systems

n/a	Involved in the study	
	☒	Antibodies
	☒	Eukaryotic cell lines
☒		Palaeontology and archaeology
☒		Animals and other organisms
☒		Clinical data
☒		Dual use research of concern

Methods

n/a	Involved in the study	
☒		ChIP-seq
	☒	Flow cytometry
☒		MRI-based neuroimaging

Antibodies

Antibodies used

The following antibodies were used to validate the extracellular vesicle isolation protocol; 1. APC-mouse anti-Human-CD9 Clone M-L13 (Clone M-L13 (RUO (GMP)), Cat# 341648, Lot# 0363084,BD Bioscience), 2.PE-labelled antihuman-CD63 (Clone H5C6 (RUO), Cat# 557305, Lot# 9143803, BD Biscience), 3. PE labeled mouse IgG1, kappa isotype control (Clone MOPC-21 (RUO), cat# 556650, Lot# 1060595, BD Bioscience) and 4. APC labeled mouse IgG1, Kappa isotype control (Clone MOPC-21 (RUO), Cat# 550854, Lot# 1032230), BD Bioscience) 5. BRIC 256 Anti-235a/GYPA- FITC (Cat# 9415FI, Lot# 3070L, IBGRL RESEARCH PRODUCTS)

Validation

Validation and technical information for all antibodies are available from the manufacturer's website as indicated below:
1. BD™ APC Mouse Anti-Human CD9 (https://www.bdbiosciences.com/en-fi/products/reagents/flow-cytometry-reagents/clinical-discovery-research/single-color-antibodies-ruo-gmp/apc-mouse-anti-human-cd9.341648). 2. BD Pharmingen™ PE Mouse Anti-Human CD63 (https://www.bdbiosciences.com/en-de/products/reagents/flow-cytometry-reagents/research-reagents/single-color-antibodies-ruo/pe-mouse-anti-human-cd63.557305). 3.BD Pharmingen™ PE Mouse IgG1, κ Isotype Control (https://www.bdbiosciences.com/en-ca/products/reagents/flow-cytometry-reagents/research-reagents/flow-cytometry-controls-and-lysates/pe-mouse-igg1-isotype-control.556650), 4. BD Pharmingen™ APC Mouse IgG1 κ Isotype Control (https://www.bdbiosciences.com/en-ca/products/reagents/flow-cytometry-reagents/research-reagents/flow-cytometry-controls-and-lysates/apc-mouse-igg1-isotype-control.550854) and 5. BRIC 256 anti CD235a/GYPA -FITC (https://nhsbtdbe.blob.core.windows.net/umbraco-assets-corp/6638/bric-256.pdf).

Eukaryotic cell lines

Policy information about cell lines and Sex and Gender in Research

Cell line source(s)

We used Plasmodium falciparum cell lines that had been obtained from malaria patients. They include KE01, sKE01, KE02, KE04, KE06 and Dd2.

Authentication

The Plasmodium falciparum cell lines were aunthenticated by genotyping though they were not aunthenticated at the time of data generation. However, the sequence data confirmed the authenticity of the isolates.

Mycoplasma contamination

Mycoplasma was checked using PCR before sample collection. All samples were collected from mycoplasma negative samples

Commonly misidentified lines
(See ICLAC register)

There are no commonly misidentified cell lines

Flow Cytometry

Plots

Confirm that:

☒ The axis labels state the marker and fluorochrome used (e.g. CD4-FITC).

☒ The axis scales are clearly visible. Include numbers along axes only for bottom left plot of group (a 'group' is an analysis of identical markers).

☐ All plots are contour plots with outliers or pseudocolor plots.

☐ A numerical value for number of cells or percentage (with statistics) is provided.

Methodology

Sample preparation

Bead assisted flow cytometry was used to validate our extracellular vesicle isolation protocol. The sample preparation protocol has been described in the methods section

Instrument

BD LSR Fortessa

Software

R software was used to analyze the data

Cell population abundance

Plasma derived extracellular vesicle populations contained either CD9 or CD63 or both. P. falciparum medium-sized extracellular vesicles contained GYPA

Gating strategy	First FSC and SSC were used to gate the beads bound by extracellular vesicles. Next, fluorochrome PE and APC conjugated to CD9 and CD63, respectively, were used to gate the positive population of extracellular vesicles. In parrallel FITC conjuagted to GYPA was used to gate for GYPA-positive population of extracellular vesicles

☒ Tick this box to confirm that a figure exemplifying the gating strategy is provided in the Supplementary Information.
