# Supplementary material for: Extracellular vesicles could be a putative posttranscriptional regulatory mechanism that shapes intracellular RNA levels in Plasmodium falciparum
Source: Nat Commun. 2023 Oct 13;14:6447. doi: 10.1038/s41467-023-42103-x (PMC10575976; doi:10.1038/s41467-023-42103-x)
Supplement: Supplementary file 4 — Description of Additional Supplementary Files [file 41467_2023_42103_MOESM4_ESM.pdf]

Description of additional supplementary files

Supplementary Data 1: The rhythmic parameters estimated from the RNA sequence data.

Supplementary Data 2: The comparison of the RNA abundance between the *Pf*EVs and Whole parasite (WP)
